# Supplementary material for: Casposase structure and the mechanistic link between DNA transposition and spacer acquisition by CRISPR-Cas
Source: eLife. 2020 Jan 8;9:e50004. doi: 10.7554/eLife.50004 (PMC6977970; doi:10.7554/eLife.50004)
Supplement: Supplementary file 1. — Random oligonucleotide sequences were generated using http://www.faculty.ucr.edu/~mmaduro/random.htm and were rejected only if they included a 3'-C nucleotide. Red indicates change from targ40 sequence. [file elife-50004-supp1.docx]

| Name/description | Sequence (5' to 3') | Figure |
| --- | --- | --- |
| LE31 | GCGTTGCGTTTTTTTGAGTTACCTATATCCC | 1B |
| RC | GGGATATAGGTAACTCAAAAAAACGCAACGC | 1B |
| LE17 | TGAGTTACCTATATCCC | 2A |
| RC | GGGATATAGGTAACTCA | 2A |
| LE17Pi | GGGATATAGGTAACTCA(Pi) | 2A |
| PiLE17 | TGAGTTACCTATATCCC(Pi) | 2A |
| LE24 | GGGATATAGGTAACTCAAAAAAAC | 2A |
| TR29 | GTTGCGTTTTTTTGAGTTACCTATATCCC | 3A-C |
| RC | ATAGGTAACTCAAAAAAACGCAAC | 3A-C |
| dsran17,ranss17 | CTGTTAGGCCACGATTG | 2B |
| RC | CAATCGTGGCCTAACAG | 2B |
| ranTR17 RC | AGGCCACGATTG | 2B |
| ssLE11 | ACCTATATCCC | 2C |
| ssLE8 | TATATCCC | 2C |
| ssLE6 | TATCCC | 2C |
| targ40 | CGTGCGTTCGAATCGCACCTCTCGCACTTTTTTTAATTAC | 3D,E |
| RC | GTAATTAAAAAAAGTGCGAGAGGTGCGATTCGAACGCACG | 3D,E |
| mutCGCA | CGTGCGTTCGAATCGCACCTCTGTAGCTTTTTTTAATTAC | 3D,E |
| RC | GTAATTAAAAAAAGCTACAGAGGTGCGATTCGAACGCACG | 3D,E |
| mutL-side | CGTGCGTTCGAATCGCACGAGACGCACTTTTTTTAATTAC | 3D,E |
| RC | GTAATTAAAAAAAGTGCGTCTCGTGCGATTCGAACGCACG | 3D,E |
| mutR-side | CGTGCGTTCGAATCGCACCTCTCGCACTTTTAAATTTTAC | 3D,E |
| RC | GTAAAATTTAAAAGTGCGAGAGGTGCGATTCGAACGCACG | 3D,E |
| mut12-17 | CGTGCCAAGCTATCGCACCTCTCGCACTTTTTTTAATTAC | 3D,E |
| RC | GTAATTAAAAAAAGTGCGAGAGGTGCGATAGCTTGGCACG | 3D,E |
| symTSD | TATATCCCCGCACTTAAGTGCG | 5-9 |
| ran40 | CACTATAGGACCATTCCATTCGAGCTAGGACCTAAGTACG | 3D,E |
| RC | CGTACTTAGGTCCTAGCTCGAATGGAATGGTCCTATAGTG | 3D,E |
| ssRNA17 | rUrGrArGrUrUrArCrCrUrArUrArUrCrCrC | 3suppl1 |
| LE20 | TTTTGAGTTACCTATATCCC | 3F |
| RC | GGGATATAGGTAACTCAAAA | 3F |
| RC-1 | GGATATAGGTAACTCAAAA | 3F |
| RC-2 | GATATAGGTAACTCAAAA | 3F |
| RC-3 | ATATAGGTAACTCAAAA | 3F |
| RC-4 | TATAGGTAACTCAAAA | 3F |
| RC-5 | ATAGGTAACTCAAAA | 3F |
| RC-6 | TAGGTAACTCAAAA | 3F |
| RC-7 | AGGTAACTCAAAA | 3F |
